# Prevalence of Chagas Disease in a U.S. Population of Latin American Immigrants with Conduction Abnormalities on Electrocardiogram

**DOI:** 10.1371/journal.pntd.0005244

**Published:** 2017-01-05

**Authors:** Mahmoud I. Traina, Salvador Hernandez, Daniel R. Sanchez, Jalal Dufani, Mohsin Salih, Adieb M. Abuhamidah, Wilman Olmedo, Jason S. Bradfield, Colin J. Forsyth, Sheba K. Meymandi

**Affiliations:** 1 Center of Excellence for Chagas Disease, Olive View-UCLA Medical Center Sylmar, CA, United States of America; 2 Division of Cardiovascular Diseases, Montefiore Medical Center/Albert Einstein College of Medicine, New York, NY, United States of America; 3 UCLA Cardiac Arrhythmia Center, Ronald Reagan UCLA Medical Center, Los Angeles, CA, United States of America; US Food and Drug Administration, UNITED STATES

## Abstract

Chagas disease (CD) affects over six million people and is a leading cause of cardiomyopathy in Latin America. Given recent migration trends, there is a large population at risk in the United States (US). Early stage cardiac involvement from CD usually presents with conduction abnormalities on electrocardiogram (ECG) including right bundle branch block (RBBB), left anterior or posterior fascicular block (LAFB or LPFB, respectively), and rarely, left bundle branch block (LBBB). Identification of disease at this stage may lead to early treatment and potentially delay the progression to impaired systolic function. All ECGs performed in a Los Angeles County hospital and clinic system were screened for the presence of RBBB, LAFB, LPFB, or LBBB. Patients were contacted and enrolled in the study if they had previously resided in Latin America for at least 12 months and had no history of cardiac disease. Enzyme-linked immunosorbent assay (ELISA) and immunofluorescence assay (IFA) tests were utilized to screen for *Trypanosoma cruzi* seropositivity. A total of 327 consecutive patients were screened for CD from January 2007 to December 2010. The mean age was 46.3 years and the mean length of stay in the US was 21.2 years. Conduction abnormalities were as follows: RBBB 40.4%, LAFB 40.1%, LPFB 2.8%, LBBB 5.5%, RBBB and LAFB 8.6%, and RBBB and LPFB 2.8%. Seventeen patients were positive by both ELISA and IFA (5.2%). The highest prevalence rate was among those with RBBB and LAFB (17.9%). There is a significant prevalence of CD in Latin American immigrants residing in Los Angeles with conduction abnormalities on ECG. Clinicians should consider evaluating all Latin American immigrant patients with unexplained conduction disease for CD.

## Author Summary

Chagas disease (CD) affects an estimated 300,000 people in the United States, but warning signs for the disease have not been closely studied. CD is usually acquired in Latin America, and can remain in the body for years or decades without producing any symptoms. However, in about 30% of patients, it can eventually result in heart failure and death. The electrocardiogram can detect potential heart problems before patients begin to feel symptoms, providing an early warning. If patients with CD receive monitoring and treatment in time, it may prevent the development of more serious heart problems. We checked for the presence of CD in a sample of 327 patients with abnormal electrocardiogram readings, all of whom had resided in Latin America for at least 12 months. Seventeen patients, or 5.2% of the total sample, were positive for CD. Our study discusses the association of different electrocardiogram readings with CD in the United States, and explores variations based on patients’ gender and country of origin. The electrocardiogram can be a valuable tool for detecting and measuring the progression of CD in patients from Latin America so that proper treatment can be offered.

## Introduction

Chagas disease (CD), caused by the protozoan *Trypanosoma cruzi*, is a slow-progressing, multi-organ disease endemic to Latin America. There are an estimated 6 million infected individuals worldwide.[1, 2] CD has an acute and chronic phase, with the chronic phase beginning 4–8 weeks after the initial infection.[3] The chronic phase begins in an asymptomatic indeterminate form characterized by seropositivity for antibodies against *T*. *cruzi*, a normal electrocardiogram (ECG), and a normal chest radiograph. Without treatment, at least 30–40% of patients with the indeterminate form will develop an advanced or determinate form 10–30 years after the initial infection.[3, 4] The advanced chronic form of CD can lead to irreversible cardiac damage resulting in conduction disease, apical aneurysms, cardiomyopathy, and sudden cardiac death. [4]

CD is traditionally associated with endemic regions in Latin America. However, given migration trends, there has been increasing recognition of populations with CD in Europe and the United States. A recent meta-analysis of European studies, which in aggregate screened 10,000 Latin American immigrants, found a CD prevalence of 4.2%.[5] Another study estimates 300,000 cases of CD in the US, contributing to 30–45,000 cases of cardiomyopathy.[1] Between 2007 and 2013, 1908 cases of CD were identified in the blood donation system.[6] In a study of blood samples in Los Angeles, 1 in 1,993 were positive for *T*.*cruzi* antibodies.[7] Nonetheless, an overwhelming majority of CD patients in the US are undiagnosed and untreated.[6, 8]

Conduction disorders are characteristic of chronic determinate Chagas disease, and are often the initial presenting finding. A study in Bolivia found ECG abnormalities in 46% of seropositive children, the most frequent being incomplete right bundle branch block (RBBB).[9] Another study in Mexico found that ECG abnormalities including RBBB were significantly higher among seropositive versus seronegative individuals.[10] In a sample of 1,389 people in a rural community of Brazil with a *T*. *cruzi* prevalence of 6.6%, ECG abnormalities were observed in 43.5% of seropositive compared with 18.3% of seronegative individuals.[11] Further, ECG abnormalities can help identify patients who are at higher risk of developing impaired systolic function. The presence of ECG abnormalities at baseline was a significant predictor of decrease in left ventricular ejection fraction (LVEF) after 17 months of follow-up in a cohort of Brazilian patients.[12]

Conduction abnormalities and cardiomyopathy are also strongly associated with CD in Latin American immigrants in the United States and Europe. In our center in Los Angeles, among adult Latin American patients with nonischemic cardiomyopathy, defined as an LVEF <40%, we found a CD prevalence of 19.2%.[13] Another study in New York identified five seropositive cases among 39 immigrants from CD-endemic countries with dilated cardiomyopathy, a prevalence of 13%.[14] Among a sample of 17 *T*. *cruzi*-positive blood donors in southeast Texas, 7 (41%) exhibited evidence of cardiomyopathy on electrocardiograph.[15] In Spain, an investigation of 485 *T*. *cruzi*-positive immigrants, of whom 459 (94.6%) were Bolivian, determined 31.5% had at least one ECG abnormality.[16] The purpose of this study is to assess the prevalence of CD in a population of Latin American immigrants with conduction abnormalities on electrocardiogram in a Los Angeles County Hospital.

## Methods

Olive View-UCLA Medical Center is a 377-bed Los Angeles County Hospital which serves a population of 2.1 million people within a catchment area of 999 square miles. Forty percent of this population is Hispanic/Latino. In 2013, nearly 10% of residents earned less than 200% of the federal poverty level, and 27% of adults (ages 18–64) were uninsured all or part of the year.[17]

All ECGs performed as part of regular clinical care at Olive View-UCLA Medical Center and three affiliated clinics between January 2007 and December 2010 were reviewed. This included ECGs for preoperative or routine examinations and patients who presented with non-specific clinical complaints such as chest pain, palpitations or shortness of breath. Enrollment criteria were: age 18–60 years old; an ECG with evidence of RBBB, LBBB, LAFB, and/or LPFB; and history of residence in Latin America for at least 12 months. All ECGs were examined for evidence of conduction abnormalities and classified by two board-certified cardiologists blinded to the study, with discrepancies resolved by a third board-certified cardiologist with consensus opinion. Duration of residency in country of origin and US were determined by interview/questionnaire. Exclusion criteria were: any known history of cardiac disease, including coronary artery disease, valvular heart disease, or cardiomyopathy, defined as LVEF ≤40%.

A total of 399 subjects were identified and met enrollment criteria: 67 subjects could not be successfully contacted and 5 subjects refused participation, resulting in a final study size of 327. A single 5 mL blood sample was obtained from all patients for *T*. *cruzi* serology testing, and a questionnaire regarding demographic information was completed at the time of study enrollment. Serological testing was performed through the Centers for Disease Control and Prevention (CDC). All samples underwent Enzyme-Linked Immunosorbent Assay (ELISA, Chagatest ELISA recombinant v. 3.0, Wiener Laboratories, Argentina) and Immunofluorescence Assay (IFA). Subjects were considered seropositive for CD only if both assays resulted positive.

### Statistical Analysis

We computed frequencies and proportions for categorical variables, and means and standard deviations for continuous variables. Chi-square tests for independence or Fisher’s exact tests, as appropriate, were used to detect associations between categorical variables, and t-tests were employed for continuous variables. All p values are two-sided, with p < 0.05 considered significant for all analyses. Analyses were conducted with SPSS software, version 23 (SPSS Inc., Chicago IL).

### Ethics Statement

The study was approved by the Institutional Review Board at Olive View-UCLA Medical Center. All participants provided written informed consent prior to participating. There was no compensation for participation.

## Results

Study participants had a mean age of 46.3±10.8 years and had resided in the U.S. for a mean of 21.3±10.7 years (Table 1). Countries of origin for the study sample were Mexico (n = 197, 60.2%), El Salvador (n = 70, 21.4%), Guatemala (n = 31, 9.5%) and other (n = 29, 8.9%: Honduras 6, Peru 6, Nicaragua 5, Argentina 5, Costa Rica 2, Colombia 2, Bolivia 2, and Chile 1). Conduction abnormalities among the study group were as follows: RBBB 40.4%, LAFB 40.1%, LPFB 2.8%, LBBB 5.5%, RBBB and LAFB 8.6%, and RBBB and LPFB 2.8% (Table 2).

**Table 1:** Baseline Characteristics, Los Angeles Patients with Conduction Abnormalities on ECG with History of at Least 12 Months Residence in Latin America.

Characteristic	*T*. *cruzi* positive	*T*. *cruzi* negative	Total	*T*. *Cruzi* prevalence	P Value*
	(n = 17)	(n = 310)	(N = 327)	(%)	
Female sex	12 (70.6)	140 (45.2)	152 (46.5)	7.9	
Male sex - no.^†^ (%)	5 (29.4)	170 (54.8)	175 (53.5)	2.9	0.048
Mean age - years (±SD)	50.8 (±10.7)	46.0 (±10.8)	46.3 (±10.8)	-	0.08
Country of origin					
Mexico - no. (%)	5 (29.4)	192 (61.9)	197 (60.2)	2.5	0.01
El Salvador - no. (%)	8 (47.1)	62 (20.0)	70 (21.4)	11.4	0.01
Guatemala - no. (%)	2 (11.8)	29 (9.4)	31 (9.5)	6.5	0.67
Other - no. (%)	2 (11.8)	27 (8.7)	29 (8.9)	6.9	0.65
Location in country of origin^ǂ^					
Rural - no. (%)	10 (58.8)	147 (48.2)	157 (48.8)	6.4	0.46
Urban - no. (%)	6 (35.3)	153 (50.2)	159 (49.4)	3.8	0.32
Rural/urban - no. (%)	1 (5.9)	5 (1.6)	6 (1.9)	16.7	0.28
Mean time in country of origin - years (±SD)	28.1 (±10.0)	25.1 (±11.1)	25.2 (±11.1)	-	0.28

* Fisher's exact test (categorical variables) or t-test (continuous variables).

† no. = number

ǂ 5 negative respondents had missing data for this category

Seventeen patients were positive for *T*. *cruzi* by both IFA and ELISA, resulting in an overall prevalence rate of 5.2% in this cohort of patients with unexplained conduction disease. These patients had not been previously diagnosed and were unaware they had CD. In the seropositive group, the mean age was 50.8±10.7 years with a mean time of residence in country of origin of 28.1±10.0 years. The difference in mean ages (4.8 years) between the seropositive and seronegative group was not statistically significant at the p<0.05 level (p = 0.08). A much smaller proportion of seropositive patients (n = 5, 29.4%) were male, compared with the seronegative group (n = 170, 54.8%), and this difference was significant (p = 0.048). The countries of origin of the seropositive patients were as follows (prevalence within subgroup in parentheses): El Salvador 8 (11.4%), Mexico 5 (2.5%), Guatemala 2 (6.5%), and other 2 (6.9%) (Table 1). There was substantial variation between countries; the prevalence was significantly lower for Mexicans yet higher for Salvadorans (p = 0.001).

**Fig 1:**
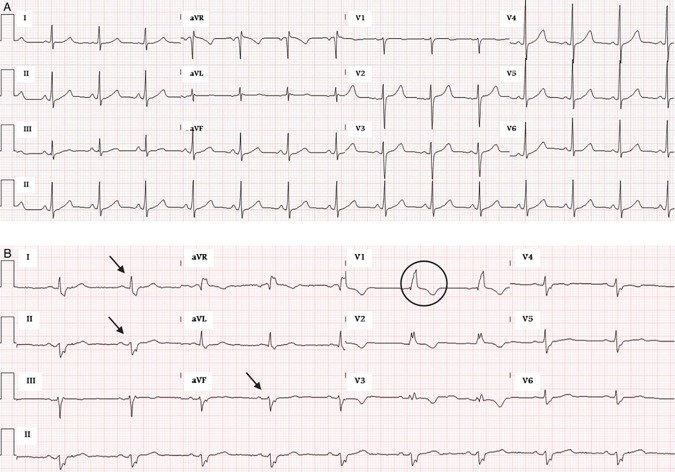
ECG examples. Panel A shows a normal ECG. Panel B is an ECG from a patient with Chagas disease; a right bundle branch block is noted in lead V1 (circle) and there is evidence of left axis deviation in the limb leads (arrows) secondary to a left anterior fascicular block. Taken together, these findings are consistent with a bifascicular block.

We found the following conduction abnormalities within the seropositive group: RBBB (n = 7, 41.2%), LAFB (n = 5, 29.4%), and RBBB in conjunction with LAFB (n = 5, 29.4%) (Fig 1, Table 2). No positive patients had LBBB, LPFB, or RBBB and LPFB. We calculated CD prevalence according to each type of conduction abnormality. For RBBB, 7/132 patients (5.3%) were seropositive, for LAFB, 5/131 (3.8%), and for RBBB/LAFB 5/28 (17.9%). The risk for positive CD diagnosis in patients with both RBBB and LAFB, compared to other conduction abnormalities in the sample, was five times greater (OR = 5.2, CI = 1.7–16.0, p = 0.002).

**Table 2:** Electrocardiographic Characteristics*.

Characteristic	*T*. *cruzi* positive	*T*. *cruzi* negative	Total	*T*. *cruzi* prevalence	P Value†
	(n = 17)	(n = 310)	(N = 327)	(%)	
RBBB - no. (%)	7 (41.2)	125 (40.3)	132 (40.4)	5.3	1.00
LAFB - no. (%)	5 (29.4)	126 (40.6)	131 (40.1)	3.8	0.45
LPFB - no. (%)	0 (0)	9 (2.9)	9 (2.8)	0	1.00
LBBB - no. (%)	0 (0)	18 (5.8)	18 (5.5)	0	0.61
RBBB and LAFB - no. (%)	5 (29.4)	23 (7.4)	28 (8.6)	17.9	0.01
RBBB and LPFB - no. (%)	0 (0)	9 (2.9)	9 (2.8)	0	1.00

* RBBB=right bundle branch block, LAFB=left anterior fascicular block, LPFB=left posterior fascicular block, LBBB=left bundle branch block.

†Fisher's exact test.

## Discussion

The majority of previous research investigations on CD-associated ECG findings have focused on populations residing in Latin America.[9, 11, 18] The prevalence of CD among Latin American immigrants in the U.S. has not been well studied. Based on immigration rates and the prevalence of *T*. *cruzi* infection in countries of origin, the CDC estimated a prevalence of 1.31% among Latin immigrants in the U.S.[1] Among a subset of Latin American-born patients with conduction abnormalities, we found a much higher *T*. *cruzi* prevalence of 5.2%. This group of patients may represent a high-risk group with the presence of early stage cardiac CD, and may benefit from closer monitoring and treatment.

Conduction abnormalities can serve as markers for early stage cardiac involvement in CD.[11, 19] Prior research has demonstrated RBBB and LAFB, isolated or in combination, to be more frequently present in CD.[9, 11] Similarly, our study found the highest prevalence in those with both RBBB and LAFB (17.9%), followed by RBBB (5.3%) and LAFB (3.8%). As expected, no patients with LBBB or LPFB had CD. The prevalence of CD among patients with both RBBB and LAFB approximates that among patients with nonischemic cardiomyopathy in an earlier study at our center.[13]

We found substantial variation by gender and country of origin. Although previous studies have noted more frequent cardiomyopathy in males with CD,[4] in our sample of patients with conduction abnormalities, there was a higher proportion of seropositive females. However, this could be due to other underlying causes for which we did not collect data. The variation in prevalence by country of residence has important clinical ramifications. The proportion of CD among those from El Salvador (11.4%) was more than twice the overall prevalence and over four times that of patients from Mexico. The WHO estimates a prevalence of CD of 1.3% in El Salvador,[2] but other evidence suggests active transmission is still disproportionately affecting some areas of the country.[20] Further, while the prevalence among Mexican patients was comparatively low in our study (2.5%), it still greatly exceeds the national prevalence estimate of 0.78.[2]

Ideally, patients born in Latin America would receive screening for presence of *T*. *cruzi* antibodies in primary care; confirmed positive cases should also undergo an ECG and other diagnostic tests to assess cardiac involvement. However, because up to 99% of people with CD are undiagnosed and *T*. *cruzi* infection is not routinely screened in the U.S. outside of blood banks,[6] it is likely many at-risk patients who present with ECG abnormalities within the medical system have not had a prior test for CD. When patients from endemic countries show ECG abnormalities characteristic of CD, it is essential to ensure they have been tested for *T*. *cruzi* antibodies so that this diagnosis can either be ruled out or utilized to inform subsequent treatment. In this study, one in five patients with bifascicular block (RBBB and LAFB) had *T*. *cruzi* infection.

Conduction abnormalities can serve as predictors of impaired systolic function in Chagas patients.[12, 18] Given the potential benefits of antiparasitic therapy at an earlier stage of CD,[21] timely identification of conduction abnormalities are an important criterion in assessing the urgency of providing treatment. Although the BENEFIT trial did not identify an advantage for antitrypanosomal therapy for patients who already had developed moderate to severely impaired systolic function, the study had a short follow-up period (5 years) and exhibited considerable intercountry variation in outcomes, which may reflect differences in *T*. *cruzi* strains.[22] The lack of advantage to treating patients with preexisting impaired systolic function in the BENEFIT trial underscores the importance of considering antitrypanosmal therapy before patients progress to the advanced form of chronic Chagas disease, especially since parasite persistence is a potential trigger of cardiac damage. [4, 23] In an Argentinian study with 21 years of follow-up, patients who received treatment with benznidazole were less likely to progress to a more severe Kuschnir classification compared to placebo.[21] Treatment with benznidazole may thus be a viable option for patients who exhibit RBBB or other conduction abnormalities yet no other signs of cardiomyopathy.

To our knowledge, no other study has retrospectively evaluated the prevalence of CD in patients with conduction abnormalities on ECG in the U.S. Our data demonstrate a significant presence of CD in this population, which is substantially higher than the proportion detected through blood sample surveillance. The presence of bifascicular block (RBBB and LAFB) and history of residence in El Salvador appear to be additional risk factors. Awareness of these potential risk factors can help focus screening to identify patients within the U.S. health system who have undiagnosed CD, so that proper treatment can be provided.

### Limitations

We did not account for potentially confounding factors such as age, diabetes mellitus, or hypertension in our analyses. The subgroup of seropositive patients was small, creating wide confidence intervals in the calculation of risk factors. Exclusion of patients with underlying cardiac disease could possibly lead to an underestimation of prevalence of CD. This study is based on a sample of patients from a Los Angeles County public hospital system; the results may not be generalizable to other locations.

## Supporting Information

S1 Checklist ECG Chagas Prevalence Strobe Checklist. (DOC)
