# Supplementary material for: Prevalence of Chagas Disease in a U.S. Population of Latin American Immigrants with Conduction Abnormalities on Electrocardiogram
Source: PLoS Negl Trop Dis. 2017 Jan 5;11(1):e0005244. doi: 10.1371/journal.pntd.0005244 (PMC5242541; doi:10.1371/journal.pntd.0005244)
Supplement: S1 Checklist — (DOC) [file pntd.0005244.s001.doc]

STROBE Statement—Checklist of items that should be included in reports of ***cross-sectional studies***

|  | Item No | Recommendation |
| --- | --- | --- |
| **Title and abstract** | 1 | (*a*) Indicate the study’s design with a commonly used term in the title or the abstract  Study of prevalence – noted in title |
|  |  | (*b*) Provide in the abstract an informative and balanced summary of what was done and what was found  “Conduction abnormalities were as follows: RBBB 40.4%, LAFB 40.1%, LPFB 2.8%, LBBB 5.5%, RBBB and LAFB 8.6%, and RBBB and LPFB 2.8%. Seventeen patients were positive by both IFA and ELISA (5.2%). The highest prevalence rate was among those with RBBB and LAFB (21.4%).  There is a significant prevalence of CD in Latin American immigrants residing in Los Angeles with conduction abnormalities on ECG.” |
| Introduction | | |
| Background/rationale | 2 | Explain the scientific background and rationale for the investigation being reported  Paragraphs 1-4 |
| Objectives | 3 | State specific objectives, including any prespecified hypotheses  “The purpose of this study is to assess the prevalence of CD in a population of Latin American immigrants with conduction abnormalities on electrocardiogram in a Los Angeles county hospital.” |
| Methods | | |
| Study design | 4 | Present key elements of study design early in the paper  Methods, Paragraphs 1 and 3 |
| Setting | 5 | Describe the setting, locations, and relevant dates, including periods of recruitment, exposure, follow-up, and data collection  “All electrocardiograms (ECGs) performed as part of regular clinical care at Olive View-UCLA Medical Center and three affiliated clinics between January 2007 and December 2010 were reviewed.” |
| Participants | 6 | (*a*) Give the eligibility criteria, and the sources and methods of selection of participants  “This included ECGs for preoperative or routine examinations and patients who presented with non-specific clinical complaints such as chest pain, palpitations or shortness of breath. Enrollment criteria were: age 18-60 years old; an ECG with evidence of RBBB, LBBB, LAFB, and/or LPFB; and history of residence in Latin America for at least 12 months.”  “Exclusion criteria were: any known history of cardiac disease, including coronary artery disease, valvular heart disease, or cardiomyopathy, defined as LVEF ≤40%.” |
| Variables | 7 | Clearly define all outcomes, exposures, predictors, potential confounders, and effect modifiers. Give diagnostic criteria, if applicable  “All ECGs were examined for evidence of conduction abnormalities and classified by two board-certified cardiologists blinded to the study, with discrepancies resolved by a third board-certified cardiologist with consensus opinion. Duration of residency in country of origin and US were determined by interview/questionnaire.”  “Serological testing was performed through the Centers for Disease Control and Prevention (CDC). All samples underwent Enzyme-Linked Immunosorbent Assay (ELISA, Chagatest ELISA recombinant v. 3.0, Wiener Laboratories, Argentina) and Immunofluorescence Assay (IFA). Subjects were considered seropositive for CD only if both assays resulted positive.” |
| Data sources/ measurement | 8* | For each variable of interest, give sources of data and details of methods of assessment (measurement). Describe comparability of assessment methods if there is more than one group |
| Bias | 9 | Describe any efforts to address potential sources of bias  “All ECGs were examined for evidence of conduction abnormalities and classified by two board-certified cardiologists blinded to the study, with discrepancies resolved by a third board-certified cardiologist with consensus opinion.” |
| Study size | 10 | Explain how the study size was arrived at  “A total of 399 subjects were identified and met enrollment criteria: 67 subjects could not be successfully contacted and 5 subjects refused participation, resulting in a final study size of 327.” |
| Quantitative variables | 11 | Explain how quantitative variables were handled in the analyses. If applicable, describe which groupings were chosen and why  “We computed frequencies and proportions for categorical variables, and means and standard deviations for continuous variables.” |
| Statistical methods | 12 | (*a*) Describe all statistical methods, including those used to control for confounding  Chi-square tests for independence or Fisher’s exact tests, as appropriate, were used to detect associations between categorical variables, and t-tests were employed for continuous variables. All p values are two-sided, with p < 0.05 considered significant for all analyses. |
|  |  | (*b*) Describe any methods used to examine subgroups and interactions  N/A |
|  |  | (*c*) Explain how missing data were addressed  N/A |
|  |  | (*d*) If applicable, describe analytical methods taking account of sampling strategy  N/A |
|  |  | (*e*) Describe any sensitivity analyses  N/A |
| Results | | |
| Participants | 13* | (a) Report numbers of individuals at each stage of study—eg numbers potentially eligible, examined for eligibility, confirmed eligible, included in the study, completing follow-up, and analysed  ““A total of 399 subjects were identified and met enrollment criteria: 67 subjects could not be successfully contacted and 5 subjects refused participation, resulting in a final study size of 327.” |
|  |  | (b) Give reasons for non-participation at each stage  Info. on reasons for refusal was not collected |
|  |  | (c) Consider use of a flow diagram |
| Descriptive data | 14* | (a) Give characteristics of study participants (eg demographic, clinical, social) and information on exposures and potential confounders  Results, Paragraph 1 |
|  |  | (b) Indicate number of participants with missing data for each variable of interest  N/A, no variables with missing data |
| Outcome data | 15* | Report numbers of outcome events or summary measures  Results, Table 2 and Paragraph 2 |
| Main results | 16 | (*a*) Give unadjusted estimates and, if applicable, confounder-adjusted estimates and their precision (eg, 95% confidence interval). Make clear which confounders were adjusted for and why they were included  Results, Table 2 and Paragraph 2 (unadjusted results) |
|  |  | (*b*) Report category boundaries when continuous variables were categorized  N/A |
|  |  | (*c*) If relevant, consider translating estimates of relative risk into absolute risk for a meaningful time period  N/A |
| Other analyses | 17 | Report other analyses done—eg analyses of subgroups and interactions, and sensitivity analyses  N/A |
| Discussion | | |
| Key results | 18 | Summarise key results with reference to study objectives  “Similarly, our study found the highest prevalence in those with both RBBB and LAFB (21.4%), followed by LAFB (4.6%) and RBBB (2.8%). As expected, no patients with LBBB or LPFB had CD.” |
| Limitations | 19 | Discuss limitations of the study, taking into account sources of potential bias or imprecision. Discuss both direction and magnitude of any potential bias  “We did not account for potentially confounding factors such as age, diabetes mellitus, or hypertension in our analyses. The subgroup of seropositive patients was small, creating wide confidence intervals in the calculation of risk factors. Exclusion of patients with underlying cardiac disease could possibly lead to an underestimation of prevalence of CD. “ |
| Interpretation | 20 | Give a cautious overall interpretation of results considering objectives, limitations, multiplicity of analyses, results from similar studies, and other relevant evidence  “Our data demonstrate a significant presence of CD in this population, which is substantially higher than the proportion detected through blood sample surveillance. The presence of bifascicular block (RBBB and LAFB) and history of residence in El Salvador appear to be additional risk factors. Awareness of these potential risk factors can help focus screening to identify patients within the U.S. health system who have undiagnosed CD, so that proper treatment can be provided.” |
| Generalisability | 21 | Discuss the generalisability (external validity) of the study results  Limitations section |
| Other information | | |
| Funding | 22 | Give the source of funding and the role of the funders for the present study and, if applicable, for the original study on which the present article is based  N/A |

*Give information separately for exposed and unexposed groups.

**Note:** An Explanation and Elaboration article discusses each checklist item and gives methodological background and published examples of transparent reporting. The STROBE checklist is best used in conjunction with this article (freely available on the Web sites of PLoS Medicine at http://www.plosmedicine.org/, Annals of Internal Medicine at http://www.annals.org/, and Epidemiology at http://www.epidem.com/). Information on the STROBE Initiative is available at www.strobe-statement.org.
